# Assessing disparities in medical students’ knowledge and attitude about monkeypox: a cross-sectional study of 27 countries across three continents

**DOI:** 10.3389/fpubh.2023.1192542

**Published:** 2023-07-26

**Authors:** Samar Abd ElHafeez, Assem Gebreal, Mohammad Adnan Khalil, Naglaa Youssef, Malik Sallam, Abdelhamid Elshabrawy, Suzan Abdel-Rahman, Amira Saad Mahboob, Saja Yazbek, Eman H. Elbanna, Riddhi Adhyaru, Zarin Nudar Rodoshi, Yap Siew Kih, Huda Jawad, Evangelos Kolotouros, Arkadiusz Jaworski, Ghadah AlQarni, Mohammad Gablan, Alexandra Condurat, Ahmed El-Sayed Said Nour Elden, Oumayma Bennani, Kamna Rawat, Areeba Ismail, Yasser Al-Hajj, Nafisa M. K. Elehamer, Jasleen Nagi, Habtamu Admassu, Saja Hassan Al Asaad, Ruthwik Duvuru, Olaoluwaposi Ogunlana, Bandar Alosaimi, Ramy Mohamed Ghazy

**Affiliations:** ^1^Department of Epidemiology, High Institute of Public Health, Alexandria University, Alexandria, Egypt; ^2^Faculty of Medicine, Alexandria University, Alexandria, Egypt; ^3^Department of Basic Medical Sciences, Faculty of Medicine, King Fahad Medical City, Riyadh Second Health Cluster, Riyadh, Saudi Arabia; ^4^Department of Medical-Surgical Nursing, College of Nursing, Princess Nourah Bint Abdulrahman University, Riyadh, Saudi Arabia; ^5^Medical-Surgical Nursing Department, Faculty of Nursing, Cairo University, Cairo, Egypt; ^6^Department of Pathology, Microbiology and Forensic Medicine, School of Medicine, The University of Jordan, Amman, Jordan; ^7^Department of Clinical Laboratories and Forensic Medicine, Jordan University Hospital, Amman, Jordan; ^8^Department of Biostatistics and Demography, Faculty of Graduate Studies for Statistical Research, Cairo University, Cairo, Egypt; ^9^Tropical Health Department, High Institute of Public Health, Alexandria University, Alexandria, Egypt; ^10^Faculty of Public Health, Lebanese University, Beirut, Lebanon; ^11^David Tvildiani Medical University, Tbilisi, Georgia; ^12^Mymensingh Medical College and Hospital, Mymensingh, Bangladesh; ^13^Taylor’s University School of Medicine, Subang Jaya, Malaysia; ^14^College of Health and Sport Sciences, University of Bahrain, Manama, Bahrain; ^15^Faculty of Medicine, National and Kapodistrian University of Athens, Athens, Greece; ^16^Faculty of Medicine, Wroclaw Medical University, Wrocław, Poland; ^17^College of Medicine, King Faisal University, Al-Ahsa, Saudi Arabia; ^18^Faculty of Medicine, Yarmouk University, Irbid, Jordan; ^19^University of Medicine and Pharmacy "Grigore T. Popa", Iași, Romania; ^20^Department of Basic Medical and Dental Sciences, Faculty of Dentistry, Zarqa University, Zarqa, Jordan; ^21^Faculty Mohammed VI of Medicine and Pharmacy, Oujda, Morocco; ^22^Government Doon Medical College, Dehradun, India; ^23^Department of Medicine, Jinnah Sindh Medical University, Karachi, Pakistan; ^24^Faculty of Medicine, Beirut Arab University, Beirut, Lebanon; ^25^Faculty of Public and Environmental Health, University of Khartoum, Khartoum, Sudan; ^26^Faculty of Medicine, Imperial College London, London, United Kingdom; ^27^Addis Ababa University CMHS, Addis Ababa, Ethiopia; ^28^Faculty of Medicine, Syrian Private University, Damascus, Syria; ^29^College of Medicine, Mohammed Bin Rashid University, Dubai, United Arab Emirates; ^30^College of Medicine, University of Ibadan, Ibadan, Nigeria; ^31^Research Center, King Fahad Medical City, Riyadh Second Health Cluster, Riyadh, Saudi Arabia

**Keywords:** monkeypox virus, knowledge, attitude, medical students, validated questionnaire

## Abstract

**Background and aims:**

The recent monkeypox (Mpox) outbreak confirmed by the World Health Organization (WHO) underscores the importance of evaluating the knowledge and attitude of medical students toward emerging diseases, given their potential roles as healthcare professionals and sources of public information during outbreaks. This study aimed to assess medical students’ knowledge and attitude about Mpox and to identify factors affecting their level of knowledge and attitude in low-income and high-income countries.

**Methods:**

A cross-sectional study was conducted on 11,919 medical students from 27 countries. A newly-developed validated questionnaire was used to collect data on knowledge (14 items), attitude (12 items), and baseline criteria. The relationship between a range of factors with knowledge and attitude was studied using univariate and multivariate analyses.

**Results:**

46% of the study participants were males; 10.7% were in their sixth year; 54.6% knew about smallpox; 84% received the coronavirus disease 2019 (COVID-19) vaccine; and 12.5% had training on Mpox. 55.3% had good knowledge of Mpox and 51.7% had a positive attitude towards it. Medical students in their third, fifth, or sixth year high- income countries who obtained information on Mpox from friends, research articles, social media and scientific websites were positive predictors for good knowledge. Conversely, being male or coming from high-income countries showed a negative relation with good knowledge about Mpox. Additionally, a positive attitude was directly influenced by residing in urban areas, being in the fifth year of medical education, having knowledge about smallpox and a history of receiving the coronavirus disease 2019 (COVID-19) vaccine. Receiving information about Mpox from social media or scientific websites and possessing good knowledge about Mpox were also predictors of a positive attitude. On the other hand, being male, employed, or receiving a training program about Mpox were inversely predicting positive attitude about Mpox.

**Conclusion:**

There were differences in knowledge and attitude towards Mpox between medical students in low and high-income countries, emphasizing the need for incorporating epidemiology of re-emerging diseases like Mpox into the medical curriculum to improve disease prevention and control.

## 1. Introduction

Monkeypox (Mpox) is a zoonotic infection caused by the monkeypox virus belonging to the genus *Orthopoxovirus* (1). The first human case of Mpox was identified in 1970 in the Democratic Republic of Congo. Since then, there have been multiple outbreaks and sporadic cases, mostly occurring in Central and West Africa (2, 3). The first cases of Mpox reported outside Africa occurred in the United States of America in 2003 (4). The number of cases has increased dramatically and the virus has spread to many other countries (5), such as the United Kingdom (UK) (6), Israel (7), and Singapore (8). In May 2022, the World Health Organization (WHO) confirmed the first Mpox outbreak outside endemic regions involving different continents worldwide (9). The WHO reported that, as of May 3, 2023, there have been more than 87,000 laboratory-confirmed cases and 130 deaths identified from 111 countries around the world (10).

Mpox is mainly transmitted via contact with respiratory secretions, infected skin lesions, or contaminated materials (11). The incubation period of Mpox is usually between 6 to 13 days, although it can last up to 21 days (12). The spectrum of Mpox disease ranges from mild to severe and can even be fatal (13). Mpox is characterized by a febrile prodrome lasting 1–4 days, accompanied by symptoms such as headache and fatigue. This is followed by the centrifugal development of deep, well-circumscribed maculopapular, vesicular, pustular, and finally crusted scab lesions. The lesions last approximately 1–3 days at each stage and progress simultaneously (14, 15). Unlike smallpox, lymphadenopathy may develop before or during the appearance of the Mpox rash (16). Mpox can cause several complications, including vomiting and diarrhea, conjunctivitis, corneal scarring, sepsis, encephalitis, bronchopneumonia, and permanent pitted scarring secondary to bacterial superinfection (17). Standard hygienic practices, vaccination against smallpox, and the use of an antiviral agent known as Tecovirimat are effective measures for the management and control of Mpox (2).

The increasing incidence of Mpox highlights the importance of its prevention, early detection, and rapid response. WHO has stated that one of the challenges in preventing the re-emergence of Mpox is a lack of knowledge of the disease, particularly among healthcare professionals (HCPs) (18). This may hinder control programs such as vaccination, especially in highly impacted countries (19, 20). Enhancing the ability of HCPs to identify cases and improve patient management is therefore an essential feature of surveillance systems for Mpox (21). Specially trained medical doctors ought to be familiar with the epidemiology of Mpox to promptly detect, report and treat new cases and prevent its spread. However, prior studies have indicated that HCPs and general practitioners have little knowledge of Mpox (22–32).

As future HCPs, assessing the knowledge and attitude of medical students towards emerging diseases like Mpox is crucial, as they could influence the general population’s perception about a range of diseases and improve public awareness about their preventive measures. Few studies have been conducted on students in health schools to assess their knowledge and attitude toward Mpox (33–38). None of these studies have compared the knowledge and attitude of medical students between high- and low-income countries. Moreover, only one study has validated a questionnaire to assess knowledge among a small sample size of 37 participants (35). Therefore, the aim of this study is to develop and validate a questionnaire to assess the knowledge and attitude of medical students towards Mpox, including a comparison of low-income and high-income countries.

## 2. Materials and methods

The Strengthening the Reporting of Observational Studies in Epidemiology (STROBE) was followed for conducting and disseminating our study (39).

### 2.1. Study design and settings

A multinational cross-sectional study was conducted. The co-author (AG) was responsible for recruiting collaborators from a proportionate number of countries representing the four regions of the world via the Global Researcher Club—an international, voluntary, and non-profit scientific research community. Collaborators from 75 countries expressed their willingness to participate in the study, but ultimately collaborators from 43 countries were enrolled. The final list included 27 countries that were able to collect data from the required sample size. These countries were then categorized according to their Gross National Income (GNI) into low-income, lower-middle-income, high-middle income and high-income countries (40).

### 2.2. Study phases

This study was conducted in two phases:

#### 2.2.1. Phase 1: development and validation of the questionnaire to assess the knowledge and attitude of medical students towards Mpox

A group of the research team who are experienced in questionnaire development and validation held four meetings to develop the questionnaire. This phase included the following steps:

##### 2.2.1.1. Identification of constructs and items

The existing literature and previously published questionnaires related to Mpox were reviewed and an item pool was developed, to be included under the knowledge and attitude constructs (33–38).

##### 2.2.1.2. Development of the items to be included under each construct

The initial questionnaire was developed in English; it consisted of 42 items, divided into 30 items for the knowledge scale and 12 items for the attitude scale.

##### 2.2.1.3. Expert evaluation

An expert panel, consisting of five investigators (one methodologist, one healthcare professional, one tropical medicine professional, and two language professionals) assessed the questionnaire for clarity and determined whether the identified items covered the defined constructs to ensure face and content validity. After three meetings, the panel agreed to remove eight items from the knowledge scale as they overlapped with other items. The second version of the questionnaire, consisting of 34 items, was used to assess its psychometric properties. Subsequently, all 43 collaborators from the enrolled countries were invited to review the pre-final copy of the questionnaire and provide feedback.

##### 2.2.1.4. Pilot testing and cognitive interviews

A pilot test of the pre-final questionnaire was carried out. Trained members of the research team from 10 randomly selected countries (Egypt, Algeria, Ethiopia, Qatar, the United States of America, China, Pakistan, Greece, the UK, and Romania) conducted cognitive interviews with 50 intended respondents (five from each country) to evaluate their understanding of the items, readability, syntax, wording, cultural appropriateness and clarity.

##### 2.2.1.5. Testing the questionnaire’s psychometric properties

A sample of 500 medical students was identified to test the reliability and validity of the pre-final version of the questionnaire. The recruitment took place between August 1st and August 15th, 2022. Participants were enrolled from Egypt, Morocco, the United Arab Emirates, Yemen, Georgia, Greece, Nigeria, Tanzania, Malaysia, and Pakistan.

##### 2.2.1.6. Final questionnaire and score interpretation

The final version of the questionnaire was in English and divided into three sections. The first section focused on collecting socio-demographic data such as age, sex, country, residence, educational year, and work status. It also included questions about the participant’s knowledge of smallpox, their history of coronavirus disease 2019 (COVID-19) vaccination, their history of chickenpox disease, their experience of receiving training programs related to Mpox, and their sources of information about Mpox. The second section consisted of 14 items with a choice of three answers (“yes”, “no”, or “uncertain”) to evaluate the knowledge of medical students regarding Mpox. This section covered different aspects of Mpox such as the pathogen, mode of transmission, clinical picture, and preventive measures. Finally, the third section assessed the attitude of medical students towards Mpox on 12 items, each having five response options based on a Likert scale ranging from “strongly disagree” to “strongly agree”.

The knowledge items in section two were scored as follows: zero for “no”, one for “uncertain”, and two for “yes”. The maximum score for the knowledge section was 28, with a higher score indicating better knowledge. The attitude questions in section three were scored using a five-point Likert scale as follows: one point for “strongly disagree”, two points for “disagree”, three points for “neutral”, four points for “agree”, and five points for “strongly agree”. The maximum score on the attitude section was 60, a high score indicating a more positive attitude. Negatively worded questions were reverse-scored to ensure consistency with positively worded statements.

#### 2.2.2. Phase 2: assessment of the knowledge and attitude of medical students about Mpox

##### 2.2.2.1. The sample size for phase 2

There was a wide variation in the knowledge and attitude levels of medical students about Mpox based on the previous literature (33–38). Using EPI-Info version 7.2 software, we assumed that 50% of the medical students had good knowledge or a positive attitude about Mpox, with a 5% accepted degree of precision and a power of 80%. Based on these assumptions, the minimum required sample size was 384 participants from each country.

##### 2.2.2.2. Sampling technique and data collection for phase-2

The final version of the questionnaire was uploaded on Google Forms and distributed via QR code flyers or online through various social media platforms (including Facebook, WhatsApp, emails, Telegram, and Twitter) to medical students in the selected countries from September 1 to December 15, 2022. Collaborators were asked to collect the data using the same techniques to minimize information bias. Each collaborator was responsible for sharing the questionnaire with medical student groups in his or her country. A convenience snowball sampling method was used to reach the required sample size of medical students from each country by asking participants to assist the collaborators in identifying further potential research participants and distributing the questionnaire accordingly. Students were eligible for inclusion if they were enrolled in public medical schools before their internship year; those from other paramedical schools were excluded from the study.

### 2.3. Statistical analysis

#### 2.3.1. Psychometric evaluation of the questionnaire

##### 2.3.1.1. Construct validity

Construct validity represents the ‘extent to which an instrument assesses a construct of concern and is associated with evidence that measures other constructs in that domain and measures specific real-world criteria’ (41). Construct validity was determined using structural, factorial, and criterion-related validity (42).

Exploratory factor analysis (EFA) was used to determine the factor structure of the questionnaire and to identify the underlying factors/constructs of our set of 34 items (43).

Before performing the EFA, factorability was assessed using both the Kaiser–Meyer–Olkin index (KMO) test and Bartlett’s test of sphericity. The KMO statistics ranged from 0 to 1, with values closer to 1 denoting greater adequacy of factor analysis. Bartlett’s test of sphericity determines whether the variables are correlated in an identity matrix; a significant value of p associated with this test (e.g., < 0.05) indicates that factorial analysis can be used (44).

To perform the EFA, principal component analysis with varimax rotation was used (45). The number of factors to be retained was determined by the eigenvalue (>1) criteria, parallel analysis, and a scree plot (46, 47). The identification of a group of questionnaire items belonging to a “factor” was achieved through a process of “factor loading”. Question items with factor loadings (cut-off value of 0.40) were associated with a distinct factor. All items with communalities less than 0.5 were deleted from the final version of the questionnaire (43).

##### 2.3.1.2. Criterion-related validity

Convergent validity was assessed by analyzing the item-to-total scores of the scale correlation. Discriminant validity was assessed by calculating the heterotrait-monotrait ratio of correlations (HTMT). If the HTMT value was below 0.90, discriminant validity was established (48).

##### 2.3.1.3. Reliability analysis

Cronbach’s *α* was calculated for the questionnaire and the scales to assess internal consistency. As a rule of thumb, Cronbach’s *α* of 0.70 to 0.80 is considered respectable for a scale for research use and an alpha of more than 0.80 is considered very good (49).

#### 2.3.2. Data management

Quantitative variables were summarized as mean ± standard deviation (SD) for normally distributed data or median [interquartile range (IQR)] for non-normally distributed data. The data distribution was checked using visual identification of a normal distribution by QQ plot. Qualitative variables were presented as percentages and frequencies.

Knowledge and attitude scores were categorized according to the median values. Participants who scored above the median value of 20 were considered to have good knowledge, while those who scored below or equal to the median were considered to have poor knowledge. Similarly, participants who scored above the median value of 47 were considered to have a positive attitude, while those who scored below or equal to the median were considered to have a negative attitude.

Independent *t*-tests were used to compare normally distributed data and the chi-square test to determine the categorical variables between knowledge and attitude categories. For correlation analysis, Spearman’s rho test was used. To identify predictors of good knowledge and positive attitude, multilevel logistic regression models were used due to the hierarchical structure of the data (medical students nested within different countries). Two separate models were produced—one for knowledge and one for attitude—with random intercept and slope. Explanatory variables were categorized as country-level (higher level) and medical students level (lower level). Country-level variables included country income classification. Medical student variables included age, sex, education level, place of residence, work status, history of chickenpox, history of COVID-19 vaccine knowledge about smallpox, training programs on Mpox, and sources of information regarding Mpox. Additionally, medical students’ knowledge of Mpox was included as an explanatory variable in the attitude model. The maximum likelihood with the Laplace approximation method was used to estimate the effect of different explanatory variables on the probability of good knowledge and positive attitude. Model fit was assessed using log-likelihood and intraclass correlation (ICC) was computed to measure clustering within groups. The likelihood ratio test indicated a significant difference after adding random effects to the intercept models. We also calculated *I*^2^ “within-cluster” which indicates how much of the total variance is due to within-cluster heterogeneity. A multilevel logistic regression model was more appropriate for estimating the clustered observations in each country. After accepting the assumption of heterogeneity of odds across the countries, the model was conducted with the random intercept and added the explanatory variables. The likelihood ratio test was used to assess the significance of the random slope for each variable. The random slope of knowledge of smallpox, the history of chickenpox disease, and the history of COVID-19 vaccine intake were then added to the models. Adding the random slopes for these variables improved the models, as their effects differed significantly across countries. Odds ratios (OR) and 95% confidence intervals (CI) were used to present data, and statistical analyses were performed using STATA version 13 and R packages (lme4) (50). All variables with *p* < 0.05 were considered significant predictors.

### 2.4. Ethical considerations

The Ethics Committee of the Faculty of Medicine, Alexandria University, Egypt (IRB No. 00012098) approved the study, following the International Ethical Guidelines for Epidemiological Studies.

## 3. Results

### 3.1. Phase 1: psychometric evaluation of the developed questionnaire to assess the knowledge and attitude about Mpox among medical students.

The mean age of the 500 medical students who participated in this phase was 21.6 ± 2.1 years, 39.4% were males and 27.8% were in their third year of medical school.

#### 3.1.1. Construct validity

Exploratory factor analysis: the KMO measure of sampling adequacy was 0.83, which was above the recommended value of 0.60, and Bartlett’s test of sphericity was highly significant (*p* < 0.001). To determine the number of factors to be retained from the EFA, parallel analysis and a scree plot (Supplementary Figure S1) were performed. The scree plot indicated that five factors should be retained. An EFA was conducted using the five-factor model, which included three subscales assessing knowledge and two subscales assessing attitude. Principal component analysis with varimax rotation was used to calculate the factor loadings of the 34 items in the questionnaire. Eight items with communalities less than 0.5 or low factor loadings were deleted, resulting in 26 items being included in the final EFA model with factor loadings greater than or equal to 0.4 (Supplementary Table S1).

Criterion-related validity: all questionnaire items were found to have a significant correlation with the total score on each scale (*p* < 0.001) indicating good convergent validity (Supplementary Table S2). Furthermore, the HTMT correlation coefficient between the five subscales was 0.32, indicating adequate discriminant validity between the subscales.

#### 3.1.2. Reliability analysis

The internal consistency of both the knowledge and attitude scales was found to be satisfactory, with an overall Cronbach’s α of 0.79. The Cronbach’s α value for the knowledge scale was 0.74, while for the attitude scale, it was 0.79 (Supplementary Table S2).

### 3.2. Phase 2: assessment of knowledge and attitude among the medical students about Mpox

#### 3.2.1. Participants’ characteristics

**Figure 1:**
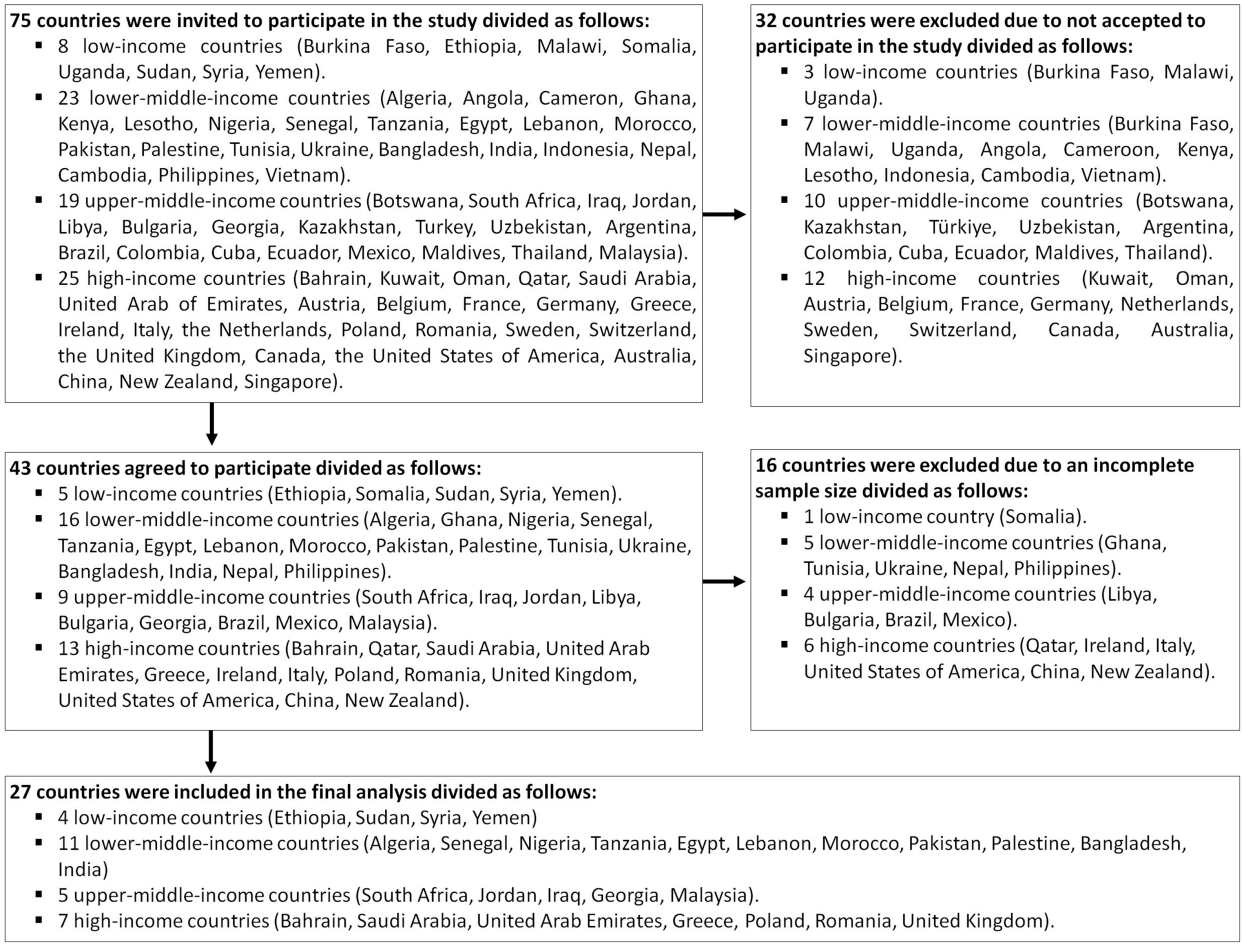
Flowchart of the included countries to assess the knowledge and attitude of medical students about human monkeypox.

Figure 1 displays the 27 countries that were included in the final analysis, with a total of 11,919 medical students. The participants had an average age of 21.7 ± 2.2 years, and 45.6% were males. Among the respondents, 21.8% were in their fourth year of medical school, 18.9% were in their third year, and 10.7% were in their sixth year. The majority (84.0%) lived in urban areas and 18% had part-time jobs. More than half of the participants (54.6%) had knowledge of smallpox, while 84.0% received COVID-19 vaccine. Among the participants, 40.9% gave a history of chickenpox infection, while 41.8% were uncertain. Only 12.5% received training programs on Mpox; the main sources of information on Mpox were social media (73.7%), scientific websites (50.6%), and friends (43.5%) (Table 1).

**Table 1:** Baseline characteristics of the study medical students (*N* = 11,919).

Characteristics	*n*	%
*Age*		
(Mean ± standard deviation)	21.7 ± 2.2	
*Sex*		
Male	5,432	45.6
Female	6,487	54.4
*Country (%)*		
Algeria	405	3.4
Bangladesh	388	3.3
Bahrain	461	3.9
Egypt	385	3.2
Ethiopia	399	3.3
Georgia	386	3.2
Greece	440	3.7
India	458	3.8
Iraq	426	3.6
Jordan	564	4.7
Lebanon	420	3.5
Malaysia	483	4.1
Morocco	573	4.8
Nigeria	402	3.4
Pakistan	745	6.3
Palestine	385	3.2
Poland	390	3.3
Romania	388	3.3
Saudi Arabia	385	3.2
Senegal	391	3.3
South Africa	385	3.2
Syria	439	3.7
Sudan	408	3.4
Tanzania	385	3.2
United Arab Emirates	480	4.0
United Kingdom	477	4.0
Yemen	471	4.0
*Educational year*		
First year	1719	14.4
Second year	2080	17.5
Third year	2248	18.9
Fourth year	2602	21.8
Fifth year	1996	16.7
Sixth year	1274	10.7
*Place of residence*		
Urban/city	10011	84.0
Rural	1908	16.0
*Employment status*		
Not-working	9178	77.0
Employed (part-time job)	2148	18.0
Employed (full time job)	593	5.0
*Knowledge of smallpox*		
Yes	6512	54.6
No	5407	45.4
*Vaccinated against COVID-19*		
Yes	10011	84.0
No	1908	16.0
*History of chickenpox disease*		
Yes	4880	40.9
No	4977	41.8
Uncertain	2062	17.3
*Receiving training programs about monkeypox*		
Yes	10428	12.5
No	1491	87.5
*Source of information about monkeypox* ^a^		
*Family members*		
Yes	4500	37.8
No	7419	62.6
*Friends*		
Yes	5183	43.5
No	6736	56.5
*Social media*		
Yes	8788	73.7
No	3131	62.7
*Research articles*		
Yes	4451	37.3
No	7468	62.7
*Scientific websites*		
Yes	6033	50.6
No	5886	49.4

a Mutually non-exclusive.

#### 3.2.2. Knowledge about monkeypox

The total knowledge score ranged from 12.7 in Bangladesh to 23.1 in South Africa (Supplementary Figure S2A). The median (IQR) total knowledge score was 20 (16–23). Medical students demonstrated good knowledge on statements where “yes” was the positive answer, such as Mpox being a viral disease (84.7%), the importance of reporting Mpox symptoms to local health authorities to prevent further transmission (75.4%), and skin rashes being a clinical manifestation of Mpox (72.4%). However, 40.8% were uncertain about the availability of a licensed Mpox vaccine at the time of the study and 32.7% were unsure about Mpox outbreaks in 2022 being related to homosexuality (Table 2).

**Table 2:** Knowledge of medical students about monkeypox (*N* = 11,919).

Components of knowledge scale	Yes	Uncertain	No
Human monkeypox is a viral disease	84.7	10.2	5.1
Monkeypox is a re-emerging disease	54.4	28.3	17.4
Monkeypox is easily transmitted from animal-to-human through direct contact	53.9	25.8	20.3
Blood-borne transmission of monkeypox is possible	45.3	35.3	19.4
Monkeypox can be transmitted through eating food	32.7	31.1	36.2
Monkeypox outbreaks in 2022 were noted to be related to homosexuality	41.2	32.7	26.2
Skin rashes are one of clinical manifestations of monkeypox disease	72.4	13.7	14.0
Avoiding contact with wild animals (alive or dead) is essential to prevent further monkeypox transmission	60.7	23	16.4
Monkeypox could be prevented by cooking meat properly	43.0	31.9	25.1
Avoiding contact with any objects that have been in contact with sick animal can prevent spread of disease	62.9	22.1	15.0
Avoiding contact with any person that has a rash can prevent the spread of disease	67.1	19.8	13.1
Avoiding contact with any object that has been in contact with sick person can prevent spread of disease	66.8	20.4	12.8
Reporting symptoms of monkeypox to local health authorities is important to prevent further disease transmission	75.4	13.5	11.1
There was a licensed monkeypox vaccine available at the time of this study	35.1	40.8	24.1
Knowledge score, median (IQR)	20 (16–23)		

**Figure 2:**
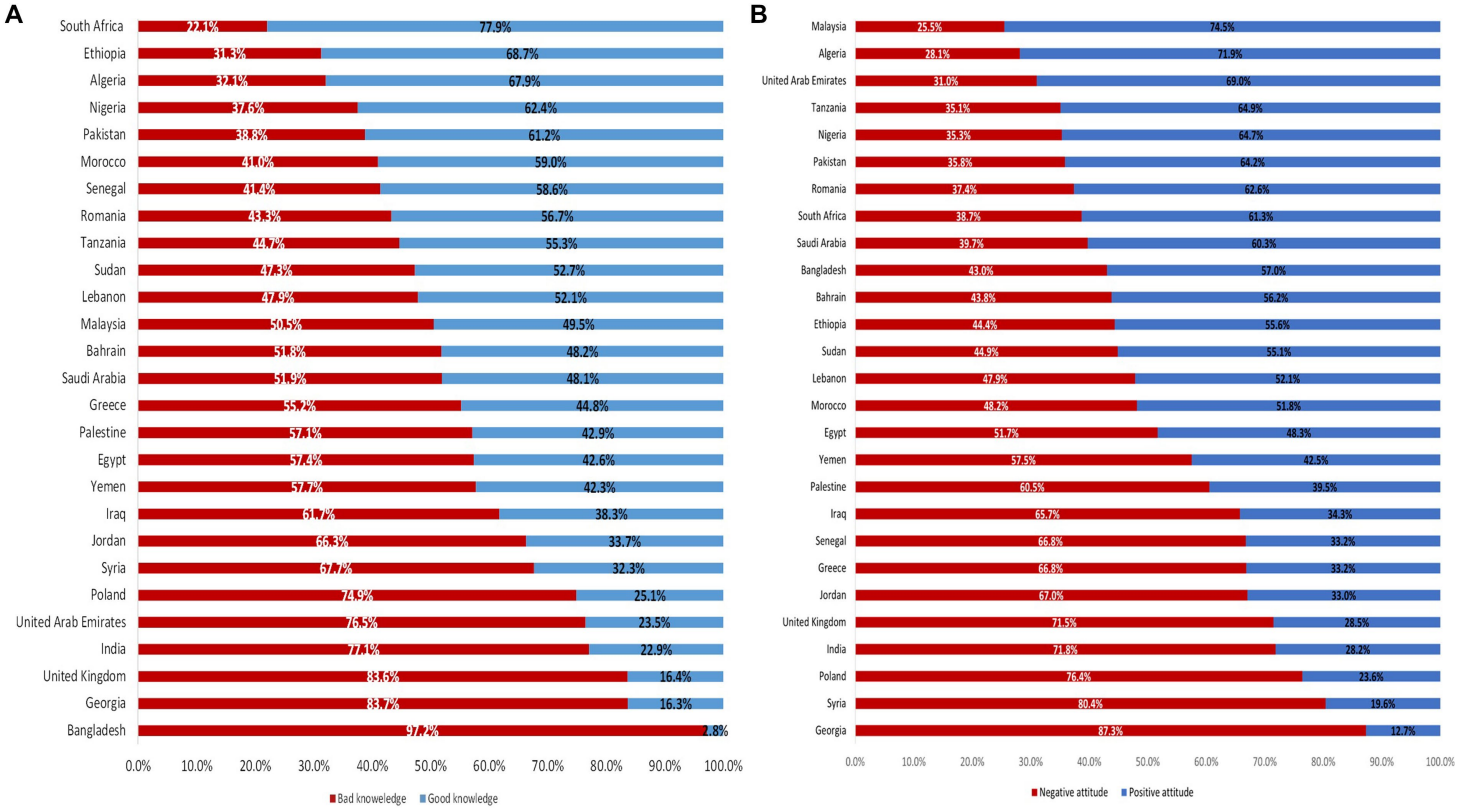
Differences between worldwide regions (low-income countries, lower-middle-income countries, upper-middle-income countries, high-income countries) in the knowledge (Figure 2A) and attitude (Figure 2B) of medical students about human monkeypox.

Overall, 55.3% of medical students had good knowledge. The lowest percentage of good knowledge was found among medical students from upper-middle-income countries (47.7%), followed by high-income countries (51.8%), while medical students from low-income countries had the highest proportion of good knowledge (53%) (Figure 2A).

#### 3.2.3. Attitude towards monkeypox

The total attitude score ranged from 40.2 in Georgia to 52.1 in Algeria (Supplementary Figure S2). The median (IQR) of the total attitude score was 47 (42–50). About two-thirds (64.3%) showed strong agreement with, “I should learn more about Mpox”; 59.3% strongly agreed that “Mpox disease prevention and control measures should be adequately available”; and 57.6% strongly agreed that, “Healthcare workers should be tested when they are in contact with someone infected”. Conversely, almost one-fourth strongly disagreed that, “I can visit any family members or friends who are infected with Mpox”, and “I do not trust the information about diseases from scientific experts”, while 21.3 strongly disagreed that, “I worry that Mpox disease is an attempt to reduce the size of the global population” (Table 3).

**Table 3:** Attitude of medical students about monkeypox (*N* = 11,919).

Components of attitude scale	Strongly agree	Agree	Neutral	Disagree	Strongly disagree
I should learn more about monkeypox disease	64.3	27.5	6.4	1.3	0.4
I worry that monkeypox disease can be transmitted to my country	39.0	32.5	21.0	5.9	1.6
Monkeypox disease prevention and control measures should be adequately available	59.3	29.2	10.1	1.2	0.2
Traveling to monkeypox disease-infected countries should be restricted	38.1	28.0	22.3	9.4	2.3
I should take monkeypox vaccine if it is available	47.0	29.0	18.4	4.4	1.2
Health care workers should be tested when they are in contact with someone infected	57.6	28.9	11.0	2.1	0.4
I can visit any family members or friends who are infected with monkeypox	14.5	14.1	20.2	27.7	23.5
I should take more hygienic preventive measures due to monkeypox disease	49.7	32.8	14.1	2.9	0.6
All people with a skin rash should be tested for monkeypox	27.4	27.3	25.8	14.2	5.4
I worry that monkeypox will become a new pandemic, and its impact will be like COVID-19	33.1	27.9	22.3	13.1	3.6
I do not trust the information about diseases from scientific experts	11.2	14.4	17.7	33.0	23.7
I worry that monkeypox disease is an attempt to reduce the size of global population	17.5	16.8	21.8	22.7	21.3
Attitude score median (IQR)	47 (43–51)				

Overall, a positive attitude was observed in 51.7% of the study participants. The lowest percentage of positive attitude was among medical students from high-income countries (29.4%), followed by upper-middle-income countries (38.9%). The highest percentage was among medical students from low-income countries (59.5%) (Figure 2B).

#### 3.2.4. Association between baseline criteria and knowledge and attitude about Mpox

The mean age of participants who had good knowledge or positive attitude (21.8 years) was significantly higher than those who had either poor knowledge or negative attitude (21.6 years). Fourth-year medical students had the highest proportion of good knowledge (22.2%) and positive attitude (23.2%). Students residing in urban areas had higher scores of good knowledge and positive attitude than others (85.1% vs. 14.9%, *p* = 0.003) and (87.6% vs. 12.4%, *p* < 0.001). In addition, students who were not working had significantly greater good knowledge and positive attitude compared to their peers (79.4% and 81.1%, respectively). Medical students who had knowledge about smallpox had higher positive attitude about Mpox compared to those who did not (57.8% vs. 42.2%, *p* < 0.001). COVID-19 vaccination was associated with a better attitude about Mpox (85.3% vs. 14.7%, *p* < 0.001). History of chickenpox infection was significantly associated with good knowledge and positive attitude about Mpox. Similar findings were observed among respondents from low-middle-income countries; they had both the good knowledge and positive attitude. Social media as a source of information was significantly associated with the highest proportion of good knowledge and positive attitude; 82.8 and 79.1% had good knowledge and positive attitude, respectively (Table 4).

**Table 4:** Association between students’ characteristics and their knowledge and attitude about monkeypox based on univariate analysis.

Baseline criteria	Knowledge			Attitude		
	Poor	Good	*p*-value	Negative	Positive	*p*-value
Age (mean ± standard deviation)	21.6 (2.2)	21.8 (2.2)	0.002	21.6 (2.2)	21.8 (2.2)	0.001
*Sex (%)*			<0.001			<0.001
Male	47.4	43.4		50.4	40.5
Female	52.6	56.6		49.6	59.5
*Educational year (%)*						
First year	15.4	13.2	<0.001	14.9	13.9	<0.001
Second year	18.8	15.7		19.7	15.0	
Third year	18.7	19.0		19.0	18.7	
Fourth year	21.6	22.2		20.5	23.2	
Fifth year	15.4	18.4		15.5	18.1	
Sixth year	10.0	11.5		10.4	11.0	
*Place of residence (%)*						
Urban/city	83.1	85.1	0.003	80.6	87.6	<0.001
Rural	16.9	14.9		19.4	12.4	
*Employment status (%)*						
Part-time job	19.2	16.6	<0.001	20.6	15.2	<0.001
Full-time job	5.8	4.0		6.2	3.6	
Not working	75.0	79.4		73.1	81.1	
*Knowledge of smallpox (%)*						<0.001
Yes	55.2	53.9	0.16	51.7	57.8
No	44.8	46.1		48.3	42.2
*History of COVID-19 vaccine intake (%)*						<0.001
Yes	83.8	84.2	0.62	82.8	85.3
No	16.2	15.8		17.2	14.7
*History of chickenpox disease (%)*						
Uncertain	19.0	15.2	<0.001	20.1	14.3	<0.001
Yes	39.3	43.0		39.1	42.9	
No	41.7	41.8		40.8	42.8	
*Receiving training programs about monkeypox (%)*						<0.001
Yes	11.8	13.3	0.02	14.8	10.0
No	882.2	86.7		85.2	90.0
*Country classification (%)*						
Low-income countries	13.4	15.6	<0.001	16.0	12.7	<0.001
Low-middle income countries	38.1	45.6		37.7	45.3	
High-middle income countries	19.5	17.9		20.6	17.0	
High-income countries	28.9	20.9		25.7	25.0	
*Source of information (%)*						
Family members						0.32
Yes	38.1	37.3	0.39	37.3	38.2
No	61.9	62.7		62.7	61.8
Friends						<0.001
Yes	41.0	46.6	<0.001	40.2	47.0
No	59.0	53.4		59.8	53.0
Social media						<0.001
Yes	66.4	82.8	<0.001	68.7	79.1
No	33.6	17.2		31.3	20.9
Research articles						<0.001
Yes	33.1	42.7	<0.001	35.8	39.0
No	66.9	57.3		64.2	61.0
Scientific websites						<0.001
Yes	45.2	57.3	<0.001	47.0	54.5
No	54.8	42.7		53.0	45.5

#### 3.2.5. Predictors of knowledge and attitude about Mpox

The study findings suggest that ICC values were 0.18 for the knowledge model with only a random intercept and 0.20 for the knowledge model with both a random intercept and random slope. *I*^2^ “within-cluster” which indicates how much of the total variance that is due to within-cluster heterogeneity. After considering the sampling variability, *I*^2^ equals 75.6% for the knowledge model. Medical students in their third year were 28% more likely to report good knowledge (OR: 1.28; 95%CI: 1.10–1.50) than those in their first year. Similarly, those in their fifth year were 45% more likely to report good knowledge (OR: 1.45; 95%CI: 1.20–1.76) and those in their sixth year were 44% (OR: 1.44; 95%CI: 1.14–1.83) more likely to report good knowledge, compared to their first-year counterparts. Furthermore, medical students who received information about Mpox from friends (OR: 1.23, 95%CI: 1.12–1.34), social media (OR: 1.67, 95%CI: 1.51–1.84), research articles (OR: 1.40; 95%CI: 1.27–1.54), and scientific websites (OR: 1.33; 95%CI: 1.21–1.46) were more likely to demonstrate good knowledge compared to those who did not receive information. However, male students had a 16% (OR: 0.84; 95%CI: 0.77–0.91) lower probability of having good knowledge about Mpox than female medical students, and those from high-income countries were 51% (OR: 0.49; 95%CI: 0.24–0.99) less likely to have good knowledge compared to students from low-income countries (Table 5).

**Table 5:** Predictors of knowledge and attitude of medical students about monkeypox based on multilevel logistic regression models.

Predictors	Knowledge	Attitude
	Odds ratio (OR) (95%CI), *p*-value	Odds ratio (OR) (95%CI), *p*-value
Age (1 year)	0.97 (0.94–1.00), 0.06	0.99 (0.96–1.02), 0.51
*sex*		
Female (reference group)	1	1
Male	0.84 (0.77–0.91), <0.001	0.72 (0.66–0.78), <0.001
*Educational year*		
First year (reference group)	1	1
Second year	1.15 (0.99–1.34), 0.06	0.89 (0.76–1.03), 0.13
Third year	1.28 (1.10–1.50), 0.002	1.08 (0.92–1.26), 0.37
Fourth-year	1.13 (0.96–1.34), 0.14	1.15 (0.97–1.37), 0.10
Fifth year	1.45 (1.20–1.76), <0.001	1.23 (1.01–1.49), 0.04
Sixth year	1.44 (1.14–1.83), 0.002	1.19 (0.94–1.51), 0.14
*Place of residence*		
Rural (reference group)	1	1
Urban/city	1.06 (0.94–1.20) 0.32	1.35 (1.20–1.53), <0.001
*Employment status*		
Not employed (reference group)	1	1
Employed (part-time job)	0.90 (0.79–1.01), 0.08	0.86 (0.77–0.98), 0.02
Employed (full time job)	0.84 (0.68–1.03), 0.09	0.66 (0.54–0.82), <0.001
*Knowledge of smallpox*		
No (reference group)	1	1
Yes	0.97 (0.76–1.25), 0.84	1.27 (1.01–1.60), 0.04
*History of COVID-19 vaccine intake*		
No (reference group)	1	1
Yes	1.17 (0.97–1.42), 0.10	1.53 (1.19–1.96), <0.001
*History of chickenpox disease*		
No (reference group)	1	1
Uncertain	0.90 (0.69–1.19), 0.47	0.81 (0.65–1.00), 0.05
Yes	1.20 (0.95–1.52), 0.12	1.10 (0.94–1.27), 0.22
*Receiving training programs about monkeypox*		
No (reference group)	1	1
Yes	0.96 (0.83–1.10), 0.56	0.74 (0.64–0.85), <0.001
*Country classification*		
Low-income countries (reference group)	1	1
Low-middle income countries	1.11 (0.58–2.13), 0.75	0.98 (0.54–1.78), 0.94
High-middle income countries	0.60 (0.28–1.26), 0.18	0.75 (0.37–1.51), 0.41
High income countries	0.49 (0.24–0.99), 0.04	0.81 (0.42–1.57), 0.53
*Source of information about monkeypox (no is the reference group)*		
Family members	0.96 (0.87–1.05), 0.38	0.98 (0.89–1.08), 0.73
Friends	1.23 (1.12–1.34) <0.001	1.09 (0.99–1.19), 0.08
Social media	1.67 (1.51–1.84) <0.001	1.29 (1.17–1.42), <0.001
Research articles	1.40 (1.27–1.54) <0.001	1.05 (0.95–1.15), 0.36
Scientific websites	1.33 (1.21–1.46) <0.001	1.21 (1.09–1.33), <0.001
*Knowledge*		
Bad knowledge (reference group)	—	1
Good knowledge		2.96 (2.71–3.23), <0.001

Regarding the attitude of medical students towards Mpox, the ICC for the model with random intercept is 0.11 and 0.17 in the case of the model including both a random intercept and random slope. The *I*^2^ equals 81.3% for the attitude model. Medical students residing in urban/city areas were 35% (OR: 1.35; 95%CI: 1.20–1.53) more likely to have a positive attitude towards Mpox compared to those who lived in rural areas, and those in the fifth year of medical education had a 23% (OR: 1.23; 95%CI: 1.01–1.49) higher probability of exhibiting a positive attitude than those in the first year. Moreover, study participants who had knowledge about smallpox were 27% (OR: 1.27; 95% CI: 1.01–1.60) more likely to show a positive attitude towards Mpox. Similarly, medical students who had received the COVID-19 vaccine had a 53% (OR: 1.53; 95%CI: 1.19–1.96) higher probability of having a positive attitude towards Mpox compared to those who did not receive the vaccine. Additionally, those who received information about Mpox from social media (OR: 1.29; 95% CI: 1.17–1.42) or scientific websites (OR: 1.21; 95% CI: 1.09–1.33) were more likely to exhibit a positive attitude towards Mpox compared to those who did not receive such information. Medical students with a good knowledge of Mpox were almost three times (OR: 2.96; 95%CI: 2.71–3.23) more likely to exhibit a positive attitude compared to those with poor knowledge. Conversely, male medical students were 28% (OR: 0.72; 95% CI: 0.66–0.78) less likely to exhibit a positive attitude towards Mpox compared to females. Furthermore, medical students who were part-time employees (OR: 0.86; 95% CI: 0.77–0.98) or full-time employees (OR: 0.66; 95% CI: 0.54–0.82) were less likely to report a positive attitude towards Mpox. Finally, medical students who received training on Mpox were also 26% (OR: 0.74; 95%CI: 0.64–0.85) less likely to exhibit a positive attitude towards Mpox (Table 5).

## 4. Discussion

This study evaluated the knowledge and attitude of 11,919 medical students from 27 low-income and high-income countries towards Mpox. In addition, the study identified factors affecting their level of knowledge and attitude about Mpox. Medical students in their third, fifth, or sixth years who accessed Mpox information from social media and scientific websites demonstrated a higher likelihood of possessing good knowledge. Conversely, being male or originating from high-income countries were linked to lower levels of knowledge regarding Mpox. Additionally, a positive attitude was directly influenced by factors such as residing in urban areas, being in the fifth year of medical education, having knowledge of smallpox, and receiving the COVID-19 vaccine. Receiving Mpox information from social media or scientific websites, along with having a solid understanding of Mpox, were also predictive of a positive attitude. On the other hand, being male, employed, or participating in a Mpox training program were inversely associated with a positive attitude towards Mpox.

The study’s findings in relation to the level of knowledge about Mpox among participants are consistent with the limited number of similar studies conducted globally. Previous findings reported disparities in knowledge about Mpox among medical students that varied from 6.3% in Pakistan (37), 22.8% in the United Arab Emirates (UAE) (35), 26% in Jordan (33), 28% in Saudi Arabia (34), and above 40% among pre-clinical and clinical dental students in Malaysia (38). This highlights the significance of this study in adding to the existing literature on Mpox knowledge among medical students and the need for more comprehensive and targeted educational efforts to improve disease prevention and control.

The diversity of the study’s results regarding the knowledge level of medical students about Mpox was analyzed. Participants from high-income countries had a low level of good knowledge about Mpox compared to low-income countries. One probable explanation for such gaps in the understanding of this growing disease is the lack of coverage of emerging viral infections, including Mpox in the country’s school health curriculum (33). Education and understanding of diseases found in those countries are sometimes relatively poor because the perception of the danger of infectious diseases’ importation and endemicity is low (51). Another explanation could be that the health of the population in low-income countries is threatened by the double burden of lifestyle-associated diseases and new and existing infectious diseases. Students in low-income countries may also have limited access to advanced medical resources, which forces them to rely on their knowledge and skills to manage different health problems, leading to a better understanding of the disease. This is the reason why there are continuous updates to medical education to train future healthcare workers in dealing with new health challenges (52).

For that, medical schools and health organizations need to consider the observed difference in knowledge levels about Mpox among medical students from different regions. They should work on developing targeted educational programs to improve knowledge and awareness of the disease. This could include incorporating Mpox into the medical curriculum, organizing training sessions, and promoting research on the disease in high-income countries. These strategies could enhance disease prevention and control globally, and lead to better preparedness in the face of future outbreaks.

A higher level of medical education, receiving information about Mpox from social media followed by research articles, scientific websites, and friends were found to impact the level of knowledge. These results find support in previous studies. For example, in the UAE and Pakistan, receiving information about Mpox was a strong determinant of good knowledge (35, 37). The re-emergence of Mpox globally has emphasized the need for different media to prioritize risk communication for zoonotic diseases using non-stop daily updates. This is expected to improve the public’s knowledge and awareness regarding Mpox (35). In contrast, male students showed low awareness about Mpox compared to female students. Findings from previous studies have also shown better knowledge among females (33, 35). Medical schools and health officials need to address the multifactorial nature of the drivers that explain the gender effect on knowledge level by tailoring the provided information. It is also worth noting that receiving information about Mpox from social media, research articles, scientific websites, and friends has an effect on the level of knowledge and highlights the importance of diverse sources of information in promoting awareness and understanding of emerging diseases. This suggests that health officials and educators should consider using multiple channels of communication to ensure that information about emerging diseases reaches as many people as possible, including medical students and healthcare professionals.

There is a dearth of studies that investigate the attitude of medical students toward Mpox. In Saudi Arabia, about 45% of medical students agreed that Mpox could transmit to their country (34), while the majority (∼90%) of pre-clinical and medical students in Malaysia had a positive attitude toward Mpox (38).

A positive attitude towards Mpox accompanied a high level of knowledge and was also predicted by achieving a high academic level, living in an urban/city environment, having knowledge about smallpox, having received the COVID-19 vaccine, and receiving information from social media or scientific websites. Conversely, male students who were employed or received training about Mpox had a negative attitude. It was speculated that this difference may be observed if assessed at the country level, as the difference might be due to women’s rights in each country and the extent to which they are allowed to be involved in education (53). Receiving training boosts medical students’ confidence and increases trust in their ability to combat the epidemic using available prevention and control measures. They are less likely to panic about new emerging diseases (54, 55). This important finding implies that educational programs and interventions aimed at improving knowledge and attitude towards Mpox need to take into account these determinants and tailor their approach accordingly. For example, such programs could use social media or scientific websites to disseminate information about Mpox to medical students. Additionally, the programs could target male students who are employed or have received training about Mpox with specific interventions to improve their attitude towards the disease.

To conclude, further research should prioritize continuous education and awareness-raising programs, along with developing strategies to address the factors that affect knowledge and attitude regarding the Mpox pandemic. This can be achieved by listening to physicians’ concerns and integrating public and health perspectives into policy and program development. It is crucial to note that a lack of knowledge about the disease can negatively impact vaccination acceptability and adherence to public health intervention strategies. Therefore, it is necessary to prioritize the education of the public and healthcare professionals to promote successful disease prevention and control.

### 4.1. Strengths and limitations

The strengths of this study were the use of a validated questionnaire to assess the knowledge and attitudes of the study participants to ensure the internal validity of the study findings. Also, we included a large sample of medical students from 27 countries across three continents, which may have enhanced the external validity of the study findings. However, this study has several limitations that should be considered when interpreting the findings. The study relied on self-reported data, which can be subject to information bias, and the sample was a convenience sample rather than a probabilistic sample. The use of electronic surveys and specific platforms may have excluded certain groups of students who did not have access to these platforms. Additionally, the timing of the survey, which was conducted during the COVID-19 pandemic, may have influenced the students’ claimed knowledge levels and might not accurately reflect the extent of instructional information supplied through university courses. In addition, cultural, religious, economic, and political differences across the study population may have influenced the knowledge and attitude of individuals towards Mpox. The study attempted to reduce this heterogeneity and ensure internal validity, external validity, and standardization of the study findings by taking the following steps. First, the use of a validated questionnaire to assess the knowledge and attitude of a large sample of medical students. It was tested by participants from 10 countries to ensure it was valid across different cultures and social backgrounds. Second, medical students who knew about infectious diseases distributed the same questionnaire and respondents completed it themselves. This ensured that all participants interpreted the questions in the same way and provided standardized responses. Finally, a large number of responses from 27 countries was collected across three continents to enhance the external validity of the study findings.

## Data availability statement

The original contributions presented in the study are included in the article/Supplementary material, further inquiries can be directed to the corresponding author.

## Ethics statement

This study was approved by the Ethics Committee of the Faculty of Medicine, Alexandria University, Egypt (IRB No. 00012098), following the International Ethical Guidelines for Epidemiological Studies. Written informed consent from the participants was not required to participate in this study in accordance with the national legislation and the institutional requirements.

## Author contributions

SA: conceptualization, methodology, validation, formal analysis, data curation, writing—original draft, writing—review and editing, visualization, and supervision. AG: validation, investigation, resources, data curation, writing—review and editing, visualization, and project administration. NY: validation, writing—review and editing, and resources. MS, BA, and MK: review and editing. AbE and SA-R: formal analysis and data curation. AM, SY, and EE: resources and validation. RA, ZR, YK, HJ, EK, AJ, GA, MG, AC, AhE, OB, KR, AI, YA-H, NE, JN, HA, SA, RD, and OO: validation and investigation. BA and RMG: conceptualization, methodology, formal analysis, writing—original draft, and writing—review and editing. All authors contributed to the article and approved the submitted version.

## Conflict of interest

The authors declare that the research was conducted in the absence of any commercial or financial relationships that could be construed as a potential conflict of interest.

## Publisher’s note

All claims expressed in this article are solely those of the authors and do not necessarily represent those of their affiliated organizations, or those of the publisher, the editors and the reviewers. Any product that may be evaluated in this article, or claim that may be made by its manufacturer, is not guaranteed or endorsed by the publisher.
