# Supplementary material for: Assessing disparities in medical students’ knowledge and attitude about monkeypox: a cross-sectional study of 27 countries across three continents
Source: Front Public Health. 2023 Jul 26;11:1192542. doi: 10.3389/fpubh.2023.1192542 (PMC10415161; doi:10.3389/fpubh.2023.1192542)
Supplement: Supplementary file 1 [file Data_Sheet_1.docx]

**Supplementary tables and figures**

**Supplementary Table 1:** Factor loadings of the different items of the knowledge and attitude scales of medical students about monkeypox (n=500).

| **Items** | **Attitude 1** | **Knowledge1** | **Knowledge 2** | **Attitude2** | **Knowledge 3** |
| --- | --- | --- | --- | --- | --- |
| **Monkeypox is a viral disease** | 0.158 | 0.171 | 0.129 | -0.299 | **0.539** |
| **Monkeypox is a bacterial disease*** | 0.022 | 0.030 | -0.258 | -0.314 | 0.161 |
| **Monkeypox disease has become a global epidemic*** | 0.152 | 0.287 | -0.068 | 0.015 | 0.321 |
| **Monkeypox is a re-emerging disease** | 0.073 | 0.073 | 0.057 | 0.026 | **0.694** |
| **Monkeypox is easily transmitted from animal-to-human through direct contact** | 0.047 | 0.133 | **0.709** | 0.077 | 0.089 |
| **Monkeypox is easily transmitted from human -to-human through direct contact*** | 0.082 | 0.248 | -0.023 | -0.031 | 0.261 |
| **Blood-borne transmission of Monkeypox is possible** | 0.068 | -0.414 | **0.564** | -0.301 | 0.003 |
| **Monkeypox can be transmitted through eating food** | -0.086 | -0.020 | **0.631** | 0.256 | 0.258 |
| **Monkeypox outbreaks in 2022 were noted to be related to homosexuality** | -0.035 | -0.294 | **0.534** | -0.208 | -0.057 |
| **The period from infection to onset of symptoms is usually from 2 - 3 weeks*** | -0.055 | -0.050 | -0.018 | -0.036 | -0.040 |
| **Skin rashes are one of clinical manifestations of human Monkeypox disease** | 0.201 | **0.611** | 0.085 | -0.146 | 0.138 |
| **Lymphadenopathy (swollen lymph nodes) is one clinical manifestations that could be used to differentiate monkeypox and smallpox cases*** | 0.092 | 0.236 | 0.026 | -0.065 | 0.370 |
| **Avoiding contact with wild animals (alive or dead) is essential to prevent further Monkeypox transmission** | 0.081 | 0.331 | **0.739** | -0.022 | -0.076 |
| **Monkeypox could be prevented by cooking meat properly** | -0.007 | 0.149 | **0.654** | 0.186 | -0.004 |
| **Avoiding contact with any objects that have been in contact with sick animal can prevent spread of disease** | 0.060 | 0.424 | **0.584** | 0.035 | -0.050 |
| **Avoiding contact with any person that has a rash can prevent the spread of disease** | 0.169 | **0.673** | 0.131 | -0.015 | 0.078 |
| **Avoiding contact with any object that has been in contact with sick person can prevent spread of disease** | 0.172 | **0.675** | 0.199 | 0.019 | -0.006 |
| **Reporting symptoms of Monkeypox to local health authorities is important to prevent further disease transmission** | 0.191 | **0.640** | 0.044 | -0.269 | 0.045 |
| **Monkeypox is usually a self-limited disease with symptoms lasting from 2 to 4 weeks*** | -0.039 | 0.247 | -0.045 | 0.126 | 0.224 |
| **Symptomatic supportive care is to be considered in the management of Monkeypox disease*** | 0.214 | 0.259 | 0.025 | -0.188 | 0.092 |
| **Monkeypox can be treated with the available antiviral medications*** | 0.014 | 0.249 | 0.019 | 0.188 | 0.293 |
| **There was a licensed Monkeypox vaccine available at the time of this study** | -0.008 | 0.099 | 0.079 | 0.159 | **0.449** |
| **I should learn more about Monkeypox disease** | **0.706** | 0.062 | 0.068 | 0.000 | 0.081 |
| **I worry that Monkeypox disease can be transmitted to my country** | **0.573** | 0.084 | 0.224 | 0.094 | -0.090 |
| **Monkeypox disease prevention and control measures should be adequately available** | **0.747** | 0.140 | -0.154 | -0.061 | 0.112 |
| **Traveling to Monkeypox disease-infected countries should be restricted** | **0.548** | 0.150 | -0.149 | 0.284 | 0.209 |
| **I should take Monkeypox vaccine if it is available** | **0.720** | 0.143 | -0.101 | 0.133 | 0.126 |
| **Health care workers should be tested when they are in contact with someone infected** | **0.778** | 0.111 | 0.118 | -0.030 | 0.040 |
| **I can visit any family members or friends who are infected with Monkeypox** | 0.159 | -0.121 | -0.187 | **0.608** | -0.029 |
| **I should take more hygienic preventive measures due to Monkeypox disease** | **0.738** | 0.226 | -0.086 | -0.008 | 0.113 |
| **All people with a skin rash should be tested for Monkeypox** | 0.410 | -0.027 | 0.188 | **0.489** | 0.140 |
| **I worry that Monkeypox will become a new pandemic, and its impact will be like COVD-19** | 0.491 | 0.079 | 0.170 | **0.435** | -0.021 |
| **I do not trust the information about diseases from scientific experts** | -0.013 | -0.068 | -0.129 | **0.704** | -0.095 |
| **I worry that Monkeypox disease is an attempt to reduce the size of global population** | 0.149 | -0.118 | 0.240 | **0.739** | -0.140 |

*Those items are deleted from the final questionnaire as their factor loadings are <0.4 on all subscales

**Supplementary Table 2:** Convergent validity and reliability of the questionnaire to assess the knowledge and attitude of medical students about monkeypox (n=500).

| Item | Components |  | Item to total score correlation* |
| --- | --- | --- | --- |
| Knowledge  Median (IQR): 20(16-23) | | | |
| MPOX_1 | Monkeypox is a viral disease | **Knowledge scale** | 0.28(<0.001) |
| MPOX_2 | Monkeypox is a re-emerging disease |  | 0.31(<0.001) |
| MPOX_3 | Monkeypox is easily transmitted from animal-to-human through direct contact |  | 0.67(<0.001) |
| MPOX_4 | Blood-borne transmission of monkeypox is possible |  | 0.28(<0.001) |
| MPOX_5 | Monkeypox can be transmitted through eating food |  | 0.58(<0.001) |
| MPOX_6 | Monkeypox outbreaks in 2022 were noted to be related to homosexuality |  | 0.32(<0.001) |
| MPOX_7 | Skin rashes are one of clinical manifestations of monkeypox disease |  | 0.32(<0.001) |
| MPOX_8 | Avoiding contact with wild animals (alive or dead) is essential to prevent further monkeypox transmission |  | 0.75(<0.001) |
| MPOX_9 | Monkeypox could be prevented by cooking meat properly |  | 0.62(<0.001) |
| MPOX_10 | Avoiding contact with any objects that have been in contact with sick animal can prevent spread of disease |  | 0.63(<0.001) |
| MPOX_11 | Avoiding contact with any person that has a rash can prevent the spread of disease] |  | 0.45(<0.001) |
| MPOX_12 | Avoiding contact with any object that has been in contact with sick person can prevent spread of disease] |  | 0.47(<0.001) |
| MPOX_13 | Reporting symptoms of monkeypox to local health authorities is important to prevent further disease transmission |  | 0.36(<0.001) |
| MPOX_14 | There was a licensed monkeypox vaccine available at the time of this study |  | 0.29(<0.001) |
| Cronbach’s alpha =0.74 | | | |
| Attitude  Median (IQR): 47(43-52) | | | |
| MPOX_15 | I should learn more about monkeypox disease | **Attitude scale** | 0.47(<0.001) |
| MPOX_16 | I worry that monkeypox disease can be transmitted to my country |  | 0.43(<0.001) |
| MPOX_17 | monkeypox disease prevention and control measures should be adequately available |  | 0.48(<0.001) |
| MPOX_18 | Traveling to monkeypox disease-infected countries should be restricted |  | 0.59(<0.001) |
| MPOX_19 | I should take monkeypox vaccine if it is available |  | 0.58(<0.001) |
| MPOX_20 | Health care workers should be tested when they are in contact with someone infected |  | 0.50(<0.001) |
| MPOX_21 | I can visit any family members or friends who are infected with Human monkeypox |  | 0.48(<0.001) |
| MPOX_22 | I should take more hygienic preventive measures due to monkeypox disease |  | 0.71(<0.001) |
| MPOX_23 | All people with a skin rash should be tested for monkeypox |  | 0.66(<0.001) |
| MPOX_24 | I worry that monkeypox will become a new pandemic, and its impact will be like COVD-19 |  | -0.55(<0.001) |
| MPOX_25 | I do not trust the information about diseases from scientific experts |  | -0.40(<0.001) |
| MPOX_26 | I worry that monkeypox disease is an attempt to reduce the size of global population |  | -0.59(<0.001) |
|  | **Cronbach’s alpha =0.79** |  |  |

**Supplementary Figure 1. Scree plot for determining the numbers of factors extracted to assess the knowledge and attitude of medical students about monkeypox.**


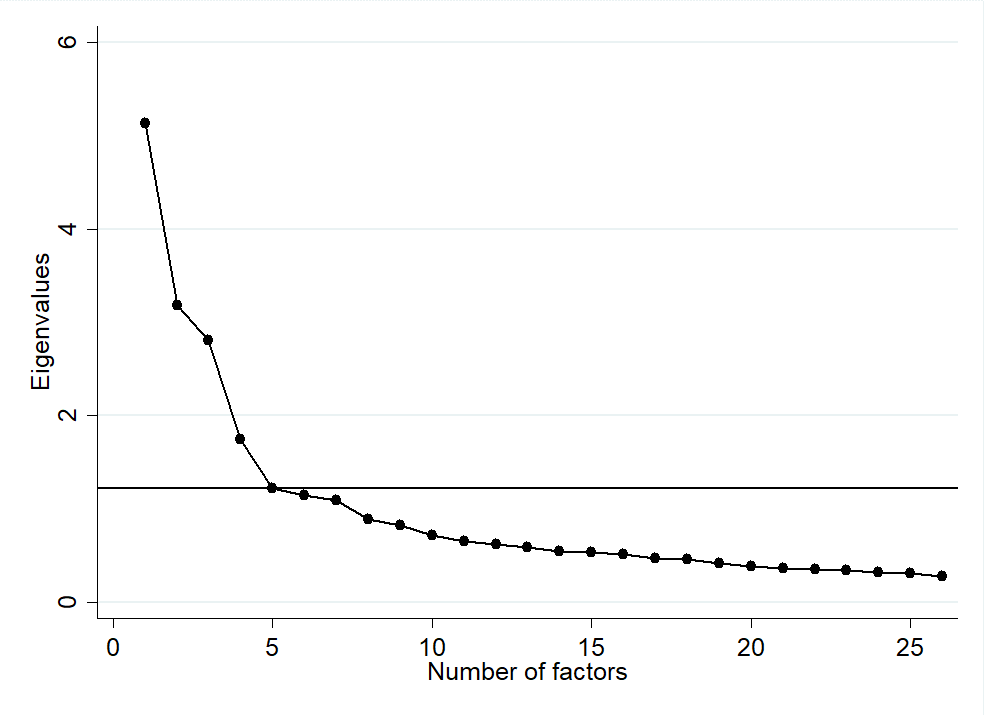


**Supplementary Figure 2. The a) knowledge and b)attitude scores of medical students about Human Monkeypox in the different countries.**

**
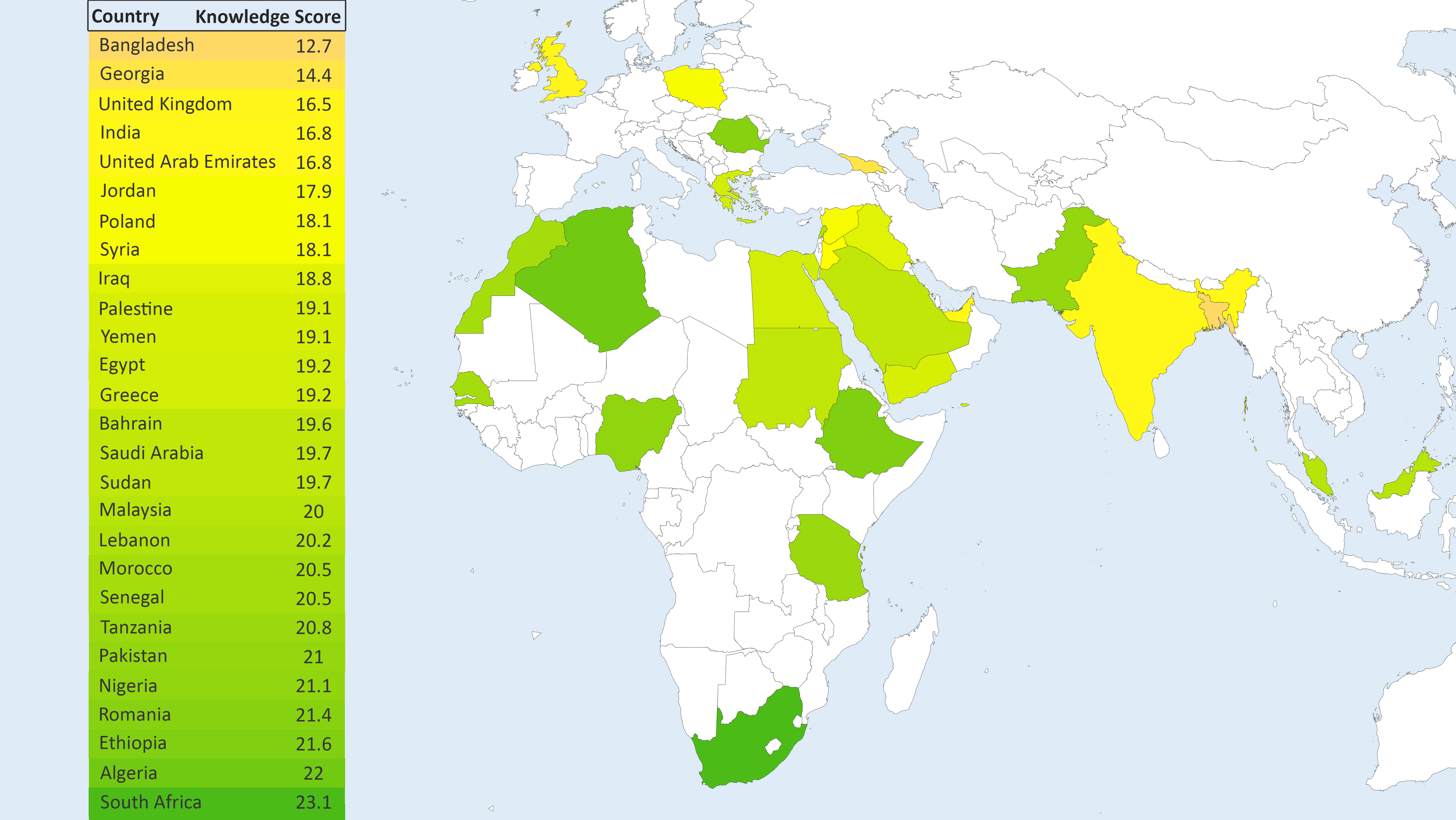
**

**(a)**


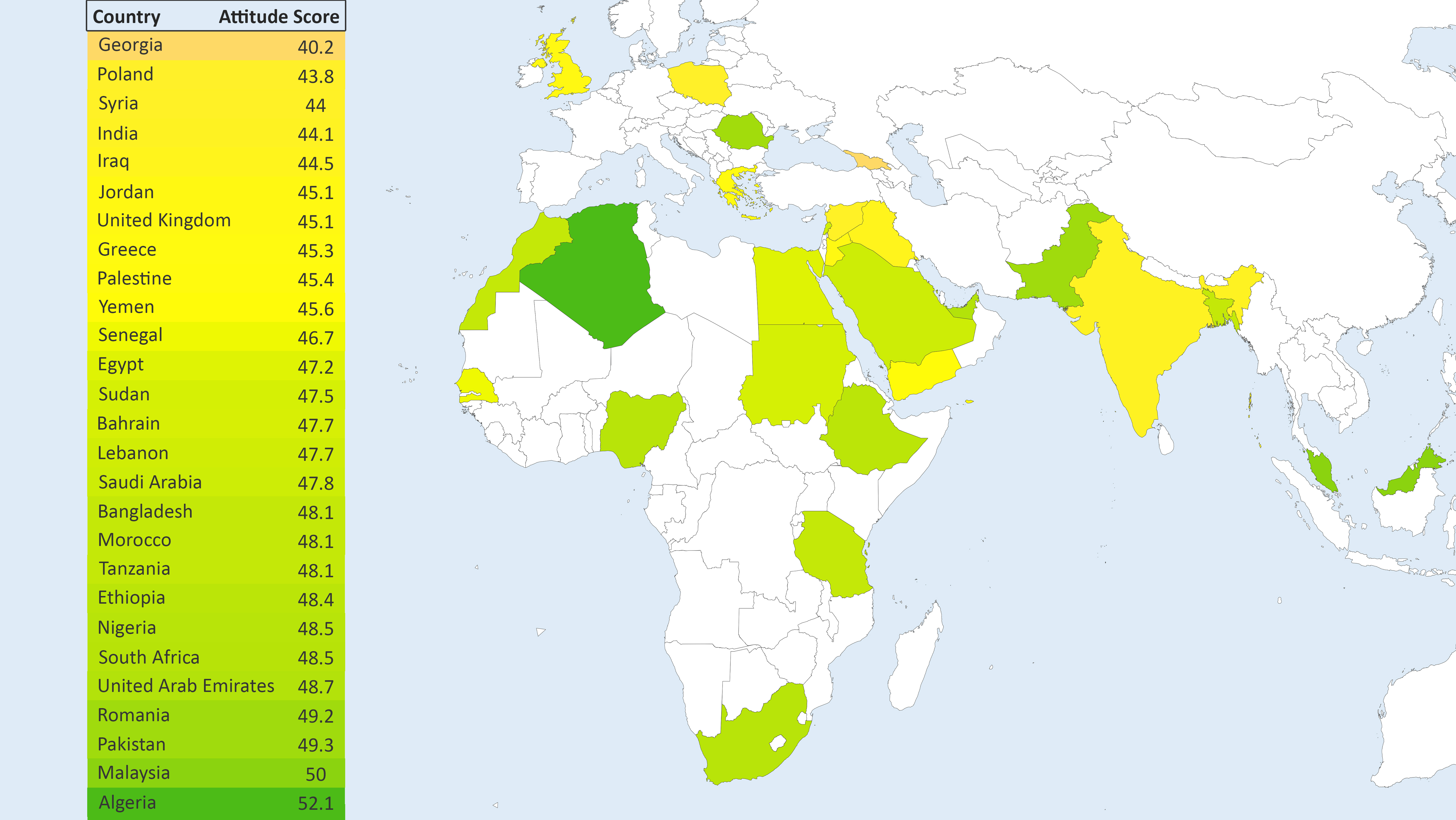


**(b**

**)**
